# Electroencephalogram Signatures of Agitation Induced by Sevoflurane and Its Association With Genetic Polymorphisms

**DOI:** 10.3389/fmed.2021.678185

**Published:** 2021-11-30

**Authors:** Shuai Zhao, Linlin Han, Ruihui Zhou, Shiqian Huang, Yafeng Wang, Feng Xu, Shaofang Shu, Leiming Xia, Xiangdong Chen

**Affiliations:** Department of Anesthesiology, Union Hospital, Tongji Medical College, Huazhong University of Science and Technology, Wuhan, China

**Keywords:** sevoflurane, anesthesia, agitation, paradoxical excitation, electroencephalogram, folate metabolism, single-nucleotide polymorphism

## Abstract

**Background:** Volatile anesthetic-induced agitation, also called paradoxical excitation, is not uncommon during anesthesia induction. Clinically, patients with agitation may lead to self-injury or disrupt the operative position, increasing the incidence of perioperative adverse events. The study was designed to investigate clinical features of sevoflurane-induced agitation and examined whether any gene polymorphisms can potentially be used to predict agitation.

**Methods:** One hundred seventy-six patients underwent anesthesia induction with sevoflurane were included in this study. Frontal electroencephalogram (EEG), electromyography (EMG), and hemodynamics were recorded continuously during anesthesia induction. DNA samples were genotyped using the Illumina Infinium Asian Screening Array and the SNaPshot technology. Genetic association was analyzed by genome-wide association study. Logistic regression analysis was used to determine the role of variables in the prediction of agitation.

**Results:** Twenty-five (14.2%) patients experienced agitation. The depth of anesthesia index (Ai index) (*p* < 0.001), EMG (*p* < 0.001), heart rate (HR) (*p* < 0.001), and mean arterial pressure (MAP) (*p* < 0.001) rapidly increased during the agitation. EEG exhibited a shift toward high frequencies with spikes during agitation. The fast waves (alpha and beta) were more pronounced and the slow rhythms (delta) were less prominent during the occurrence of agitation. Moreover, three SNPs in the methionine synthase reductase (MTRR) gene were correlated to the susceptibility to agitation (*p* < 5.0 × 10^−6^). Carrying rs1801394 A > G (odds ratio 3.50, 95% CI 1.43–9.45) and/or rs2307116 G > A (3.31, 1.36–8.95) predicted a higher risk of agitation.

**Discussion:** This study suggests that the agitation/paradoxical excitation induced by sevoflurane is characterized as increases in Ai index, EMG, HR and MAP, and the high frequency with spikes in EEG. Moreover, our results provide preliminary evidence for MTRR genetic polymorphisms, involving folate metabolism function, may be related to the susceptibility to agitation.

**Clinical Trial Number and Registry URL:** ChiCTR1900026218; http://www.chictr.org.cn/showproj.aspx?proj=40655.

## Introduction

Volatile anesthetics have been widely used for the induction and maintenance of anesthesia in clinics for more than 170 years, but the mechanism of their effects remains elusive (1). Sevoflurane is being utilized increasingly in clinic, but it also can cause adverse effects, such as induction and emergence agitation, malignant hyperthermia, nausea, and vomiting (2–4). Agitation, also called paradoxical excitation, is not uncommon during anesthesia induction with an incidence reported to range from 5 to 60% (5–7). In clinical practice, patients with agitation or involuntary body movements may lead to self-injury or disrupt the dressing, operative position, or indwelling devices, increasing the incidence of perioperative adverse events (5, 8).

Previous studies have been reported that induction agitation, emergence agitation, and postoperative delirium have similar characteristics with disturbance of consciousness and excited behavior (5, 9–11). This implies that the same or similar mechanism may underlie. Thus, characteristic changes in hemodynamics and electroencephalogram (EEG) during agitation deserve particular attention, which may facilitate further understanding of emergence agitation and postoperative delirium. Anesthesia induction with sevoflurane can induce an initial increase in heart rate (HR) and a comparable reduction in systolic blood pressure (5), however, this is distinctly different from the changes in hemodynamics during agitation. Moreover, anesthesia induction with sevoflurane is related to seizure-like electroencephalographic activity and has been reported in observational studies (7, 12, 13), but it is unclear whether epileptiform electroencephalographic activity is the same as EEG signatures during agitation.

Also, although sevoflurane, children, the female gender, speed of anesthesia induction, and high alveolar concentration have been reported as risk factors of anesthesia-induced agitation/paradoxical excitation (12, 14, 15), the underlying mechanisms remain unknown. Our clinical observations found that individuals showed difference in the susceptibility to agitation/paradoxical excitation. Therefore, we hypothesized that a genetic predisposition for agitation may exist and could explain individual variation in the susceptibility to agitation/paradoxical excitation.

Therefore, the aim of this study was to investigate clinical features of sevoflurane-induced agitation/paradoxical excitation in hemodynamics, EEG, and explore its association with gene polymorphism. Our present study may help anesthesiologists to identify agitation/paradoxical excitation during anesthesia induction in time and provide clues for further research on its mechanisms.

## Materials and Methods

### Participants

This study protocol was approved by the local ethics committee (Institutional Ethics Committee of Tongji Medical College, Huazhong University of Science and Technology, 2019-S1205) and registered at the Chinese Clinical Trial Registry with ChiCTR1900026218. All methods were conducted following the relevant regulations and guidelines of the institutional ethics committee. NIH guidelines for conducting human genetic research were followed. Participants gave informed consent for genetic testing and analysis. Informed consent was provided from all individuals before commencing study. This article adheres to the applicable CONSORT guidelines. Inclusion criteria: (1) elective surgery, (2) provided informed consent, (3) patients were 25-55 years and had a BMI of 20-30 kg/m^2^, (4) ASA I or II, and (5) no history of allergy and drug addiction. Exclusion criteria: (1) history of hepatic or renal disease, hyperthyroidism, hypothyroidism, and severe anemia; (2) severe hypertension or coronary heart disease; (3) any neurological or psychological disease; (4) infectious diseases (include viral hepatitis, syphilis).

### Clinical Protocol

All patients followed strict fasting guidelines according to the American Society of Anesthesiologists (ASA) guidelines before the procedure (16). None of the patients received premedication before anesthesia. Upon arrival in the operating room, patients were monitored with an electrocardiograph (ConView system, China), pulse oximeter, non-invasive arterial pressure (NIAP), and electrocardiography. Anesthesia was induced by sevoflurane using an anesthesia workstation (Dräger Telford, PA, USA). Three percent sevoflurane was inhaled with oxygen (3 L/min) *via* face mask. End-tidal CO_2_ and sevoflurane concentration were continuously determined with a monitor accurate to within 0.1% (Drägerwerk, Lübeck, Germany). All patients breathed spontaneously throughout the sevoflurane induction procedure. Before the administration of sevoflurane, the respiratory circuit was pre-infused with 3% sevoflurane with oxygen (3 L/min). Anesthesia was induced from purposeful movement and loss of verbal (LOV) to loss of response to trapezius squeeze (LOTS). The duration of anesthesia induction for all patients in our study was 300–600 s. The facemask was kept sealed during the anesthesia induction. Anesthesia induction, in all patients, was administered by one experienced anesthesiologist. During the induction of anesthesia, another investigator evaluated and recorded LOV and LOTS (using Extended Observer's Assessment of Alertness and Sedation) (17). All procedure was recorded by the camera with participants consent for further analysis.

After induction of anesthesia with sevoflurane, the trachea was intubated and mechanical ventilation was started after intravenous propofol, sufentanil, and cisatracurium injections by one skilled anesthetist. Subsequently, anesthesia was maintained with propofol and remifentanil. All patients had stable vital signs intraoperatively and finished the surgery successfully.

### Agitation Behavior Evaluation

The anesthesia induction period was videotaped continuously and reviewed by an independent neurologist to assess the presence of agitation. We defined the uncoordinated movements of body and/or limbs during anesthesia induction as agitation, and excluded the movements regarding mask placement.

### Electrocardiograph Recording and Analysis

The disposable sensor (Pearlcare, China) was applied to the forehead of each patient after arrived at the operating room. Then, connected it to the YY-106 ConView monitor (Pearlcare, China), which could record the raw EEG and EMG of frontal-temporal muscle signals and calculate Ai index simultaneously. The signal was acquired (500 Hz) on the ConView YY-106 monitor and processed through 0–50 Hz band-pass digital filter. The forehead skin was prepared to ensure low impedance for real-time signal recording. Before anesthesia induction, baseline EEG and Ai index were recorded with the patients lying comfortably and eyes closed in an operating theater suite. The baseline of Ai index was calculated as the mean values during a 60 s period prior to induction and the nadir of Ai index was the lowest value detected before agitation.

Ai index is an index of depth of anesthesia. It is calculated based on sample entropy (SampEn), 95% spectral edge frequency (95% SEF), and burst suppression ratio (BSR). Ai index ranges from an isoelectric EEG (0) to wake (80–99), general anesthesia (40–60), and light/moderate sedation (60–80), this feature is the same as Bispectral Index (BIS). Ai index has comparable traits of BIS and shows the advantage of SampEn for demonstrating the level of conscious, which has been validated previously in a multicenter study (18).

The unprocessed EEG data was analyzed using Welch's and/or Fast Fourier transformation (FFT) methods in MATLAB (MathWorks Inc., Natick, MA). All EEG data were reviewed by an independent blinded investigator experienced in EEG interpretation.

### DNA Sample Collection and Analysis

We collected the arterial whole blood samples (~4 ml) in the EDTA-Vacutainer tube and stored them at −80°C until processed. Preliminary DNA samples (*n* = 30, 15 patients each in the two groups) were genotyped on an Illumina Infinium Asian Screening Array microchip (Illumina Inc., San Diego, USA). The Illumina Infinium Asian Screening Array microchip contains more than 700k markers and combines genome-wide coverage of East Asian populations and relevant clinical research content. Markers with allele numbers larger than two, missing data greater than 0.5, minor allele frequency lesser than 0.05, and heterozygosity greater than 0.8 were removed resulting in 359,584 SNPs. A total of 359,584 high-quality SNPs were used to perform genome-wide association study (GWAS) for five traits in TASSEL v.5.2.54 software (19), using a simple linear model (GLM). Five traits for GWAS included age, height, weight, BMI, and susceptibility to agitation. The significance threshold of genome-wide was assessed with the formula *p* = 0.05/n, where the *n* is the effective number of independent SNPs (20). Therefore, the *p*-value thresholds for significance in our study were approximately 5 × 10^−6^. Then, all the DNA samples of the patients (*n* = 176) were genotyped by the SNaPshot technology for validation based on the results of GWAS. Linkage disequilibrium (LD) test was performed using PopldDecay v.3.40 software suite (21) base on filtered SNPs.

### Statistical Analysis

All statistical analysis was performed with GraphPad Prism version 8 (Graph-Pad Software Inc., San Diego, CA, USA), SPSS software (version 25 for Mac; IBM, New York, USA), and MATLAB (MathWorks Inc., Natick, MA). Data are expressed as Mean ± SD or median and 25th−75th percentile. The differences in age, weight, height, and BMI between two groups were determined by the independent-sample *t*-test. Differences in sex and SNPs were analyzed using Pearson Chi-square tests. Physiological parameters data (include Ai index, EMG, HR, MAP) were analyzed by repeated measures analysis of variance (Bonferroni *post hoc* test). EEG data were analyzed using non-parametric repeated measures ANOVA (Friedman's test using Bonferroni *post hoc* test). LD analysis was performed to determine the associations of different SNPs. A standardized coefficient of *R*^2^ was used to evaluate LD. The higher value of *R*^2^ indicated the stronger LD. *R*^2^ > 0.8 represented high LD. Univariable logistic regression analysis was used to determine the role of variables in the prediction of agitation. Significance was assigned at *p* < 0.05.

## Results

**Figure 1:**
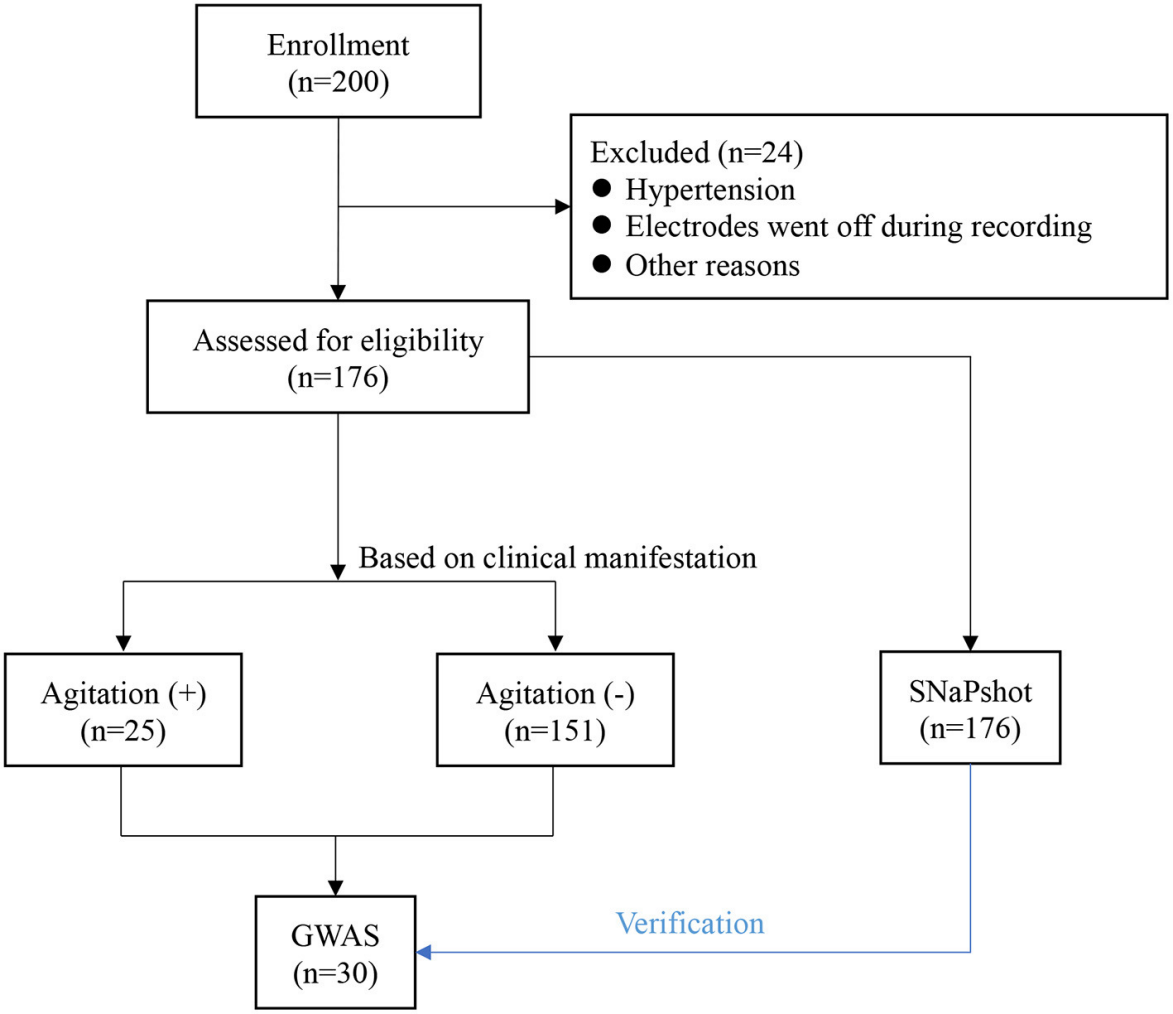
Flow chart of the study design of this study. GWAS, genome-wide association study.

**Figure 2:**
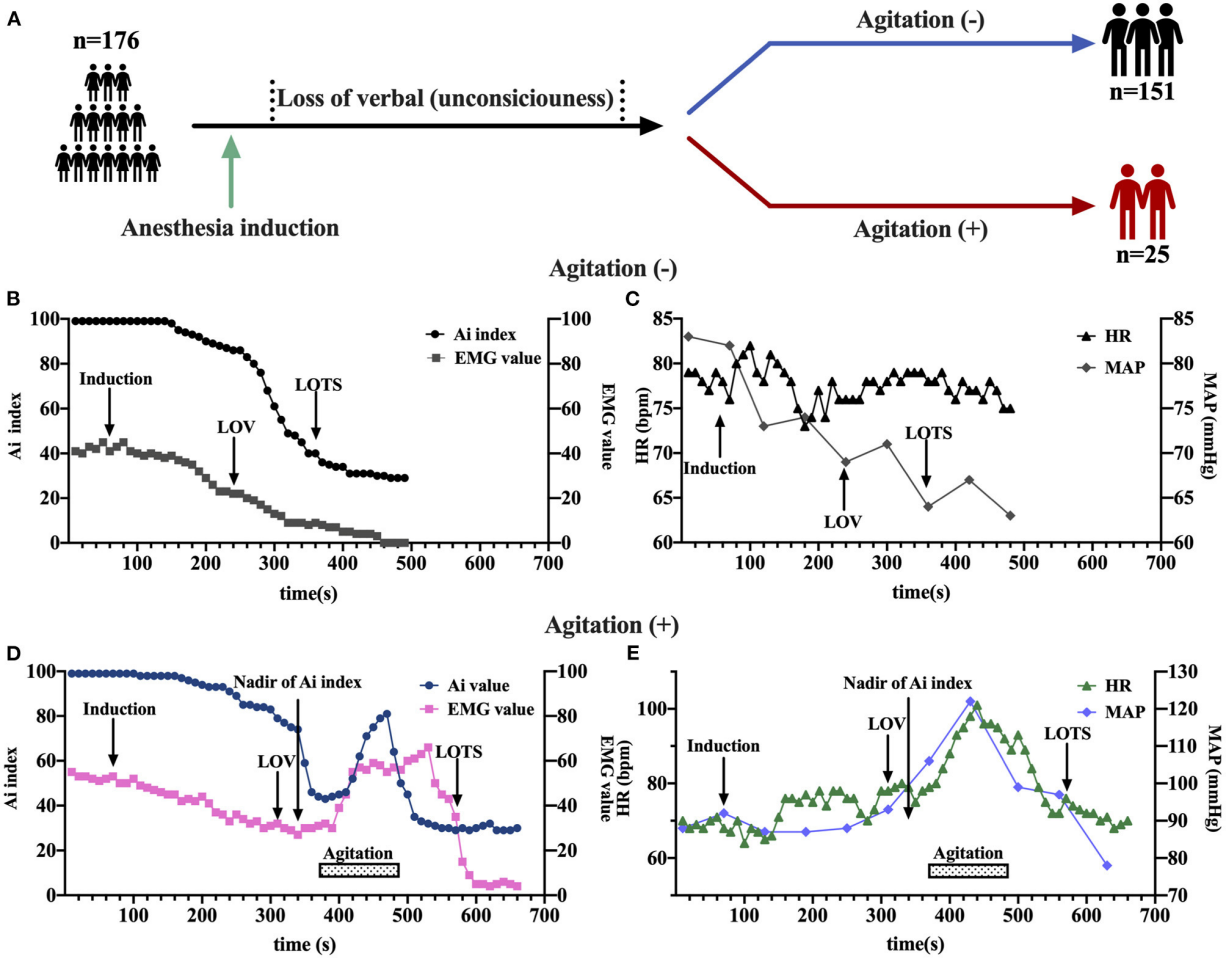
The schematic of the anesthesia induction protocol and data from two illustrative subjects. **(A)** Sevoflurane was administered to anesthesia induction. **(B–E)** Examples of time course of Ai index, electromyography (EMG), Heart rate (HR), and mean arterial pressure (MAP) during sevoflurane induction at baseline, loss of verbal (LOV), loss of response to trapezius squeeze (LOTS), and agitation behavior in two groups with either the occurrence of agitation **(D,E)** or not **(B,C)**. The LOV, LOTS, Nadir of Ai index and agitation period were shown. EMG, electromyography; Ai index, the depth of anesthesia index; MAP, mean arterial pressure; HR, heart rate; LOV, loss of verbal; LOTS, loss of response to trapezius squeeze.

In the cohort of 176 patients (81 men and 95 women), the mean (SD) age was 42.24 (10.77) years (Table 1). Of all patients, 25 (14.2%) patients experienced agitation 0.25 patients with high-quality clinical cases were enrolled in the agitation (+) group, 151 patients in agitation (-) group for control. The two groups were comparable concerning sex, age, height, weight, and body mass index (BMI) (Table 1). They also underwent the same clinical protocol and sequencing process. No adverse events were reported during this study. The flow chart of this study is shown in Figure 1. The schematic of the anesthesia induction protocol and data from two illustrative subjects were presented in Figure 2.

**Table 1:** Characteristics of included patients (*n* = 176).

	**All patients** **(***n*** = 176)**	**Agitation (+)** **(***n*** = 25)**	**Agitation (-)** **(***n***=151)**	***P*** **value**
Sex	.	.	.	0.517
Male: *n* (%)	81 (46.02)	13 (52.00)	68 (45.03)	.
Female: *n* (%)	95 (53.98)	12 (48.00)	83 (54.97)	.
Age (year)	42.24 ± 10.77	40.72 ± 10.45	42.50 ± 10.84	0.447
Height (cm)	165.31 ± 7.81	165.88 ± 8.07	165.21 ± 7.79	0.693
Weight (kg)	63.13 ± 11.00	64.68 ± 13.12	62.87 ± 10.63	0.449
BMI (kg/m^2^)	23.01 ± 3.05	23.29 ± 3.03	22.97 ± 3.06	0.620

*Age, height, weight and BMI were presented as mean ± SD. Male and female were expressed as number (percentage). No significant differences were noted. Independent-sample t-test (age, weight, height, and body mass index) and Pearson Chi-square tests (sex). p < 0.05 was considered statistically significant*.

*BMI, body mass index*.

**Figure 3:**
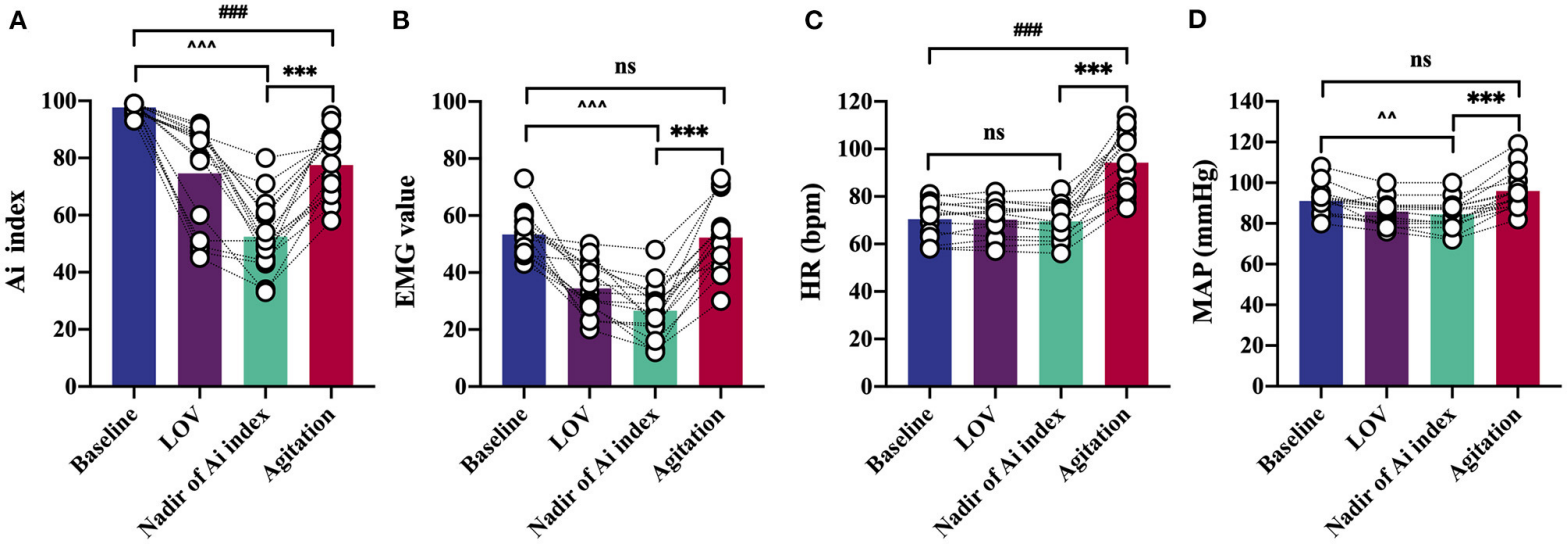
Detailed analysis of characteristic changes of the Ai index, EMG, HR, and MAP in agitation (+) group (*n* = 15). Ai index **(A)**, EMG **(B)** and MAP **(D)** decreased before agitation. Ai index, EMG, HR **(C)**, and MAP increased during agitation period. Data are expressed as mean ± SD; ^***^p < 0.001; ^###^*p* < 0.001; ^∧∧^*p* < 0.01, ^∧∧∧^*p* < 0.001; ns, not significant; Repeated measures analysis of variance using Bonferroni *post hoc* tests **(A–D)**. EMG, electromyography; Ai index, the depth of anesthesia index; MAP, mean arterial pressure; HR, heart rate; LOV, loss of verbal; LOTS, loss of response to trapezius squeeze.

Anesthesia induction was associated with a rapid decrease in Ai index. The maximal depression of Ai index (the lowest value detected before the occurrence of agitation) was defined as the nadir of Ai index in the agitation (+) group (*n* = 15). The Ai index, EMG, and MAP decreased in a depth of anesthesia manner, reaching the maximal depression at the nadir of Ai index (Figures 3A,B,D). No significant changes in HR were found before agitation (Figure 3C). It was followed by a significant and rapid increase in Ai index, EMG, HR, and MAP during the agitation period compared to the nadir of Ai index. The Ai index increased from 52 ± 13 to 78 ± 11 (*p* < 0.001), the EMG increased from 27 ± 10 to 52 ± 12 (*p* < 0.001), the HR increased from 70 ± 9 to 94 ± 14 (*p* < 0.001), and the MAP increased from 84 ± 7 to 96 ± 10 (*p* < 0.001). Then, this trend gradually decreased to the previous deep anesthesia sedation level with the disappearance of the agitation. There were no observations of similar changes in Ai index, EMG, HR, and MAP in the agitation (-) group (*n* = 15).

**Figure 4:**
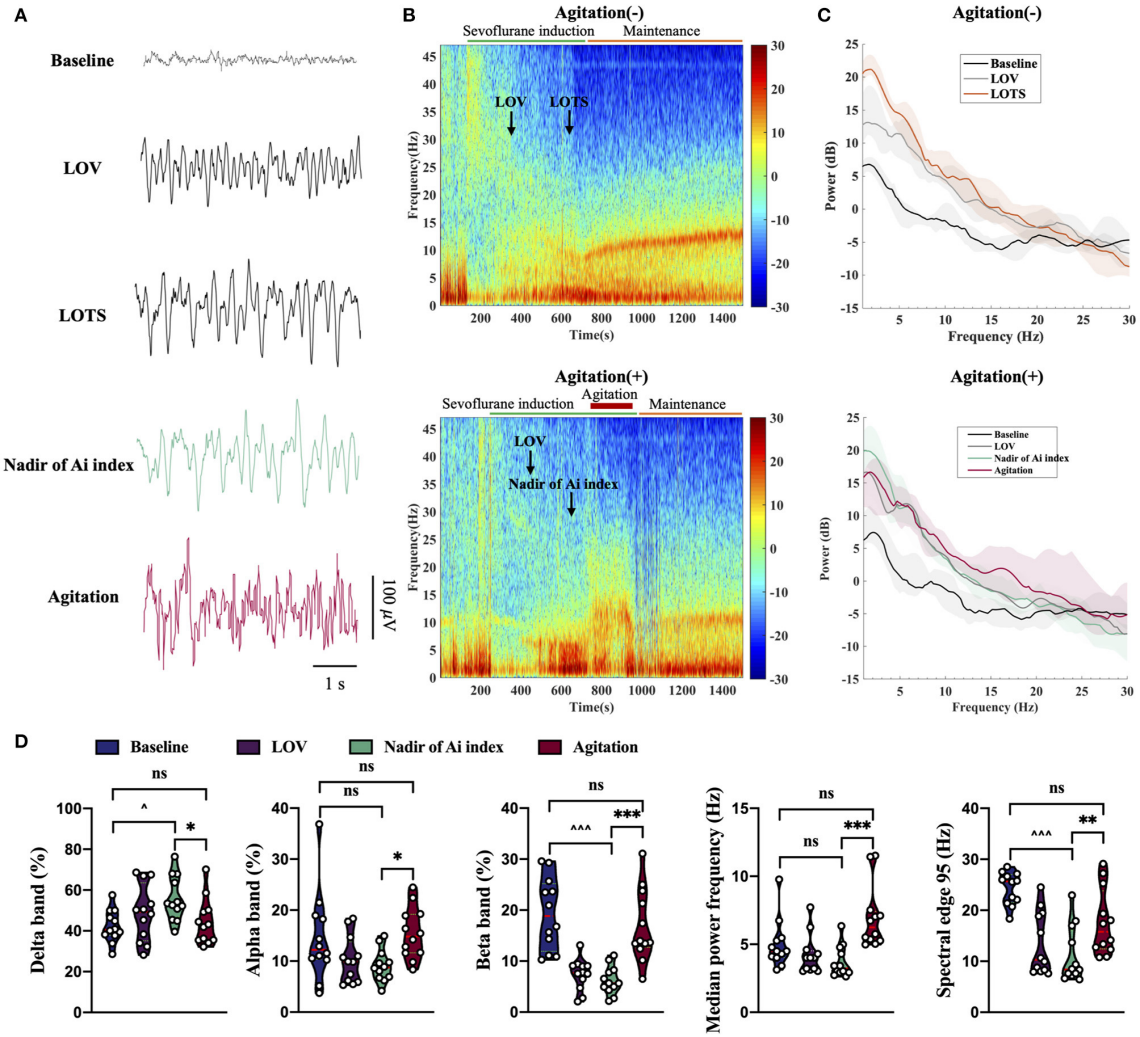
Unprocessed EEG waveform, spectrogram, spectrum and EEG analysis from agitation (+) group (*n* = 15) and agitation (-) group (*n* = 15). **(A)** Sample of 5 s EEG traces recorded during sevoflurane-induced different states. Agitation displayed a significant transition of the EEG oscillations to high frequencies with spikes. **(B)** Spectrogram shown an increase in the fast waves (alpha and beta) and a decrease in the slow rhythms (delta) during agitation. The LOV, LOTS, Nadir of Ai index, and agitation period are shown. **(C)** Spectral bands of delta (1–4 Hz), theta (4–8 Hz), alpha (8–13 Hz), and beta (13–30 Hz) were analyzed from two groups. The power spectra across the 1–30 Hz frequency band were presented (solid line, median; shaded area, 25th−75th percentile). **(D)** Changes in delta band (%), alpha band (%), beta band (%), MPF, and SEF95 during agitation. Center (red) line, median; green line, upper and lower quartiles. ^*^p < 0.05, ^**^p < 0.01, ^***^p < 0.001; ^∧^*p* < 0.05, ^∧∧∧^*p* < 0.001; ns, not significant; Non-parametric repeated measures analysis of variance (Friedman's test) using Bonferroni *post hoc* tests **(D)**. EEG, electroencephalogram; EMG, electromyography; Ai index, the depth of anesthesia index; MAP, mean arterial pressure; HR, heart rate; LOV, loss of verbal; LOTS, loss of response to trapezius squeeze; MPF, median power frequency; SEF95, spectral edge 95.

Regarding the raw EEG data, these raw waveforms underwent similar changes as the depth of anesthesia increased in both agitation (+) group (*n* = 15) and agitation (-) group (*n* = 15). The frequency was high and the amplitude was shallow during light anesthesia (LOV). The frequency slowed and amplitude deepened, when a deeper depth of anesthesia achieved (LOTS and/or Nadir of Ai index). But the wave change displayed a significant transition of the EEG oscillations to high frequencies with spikes when the agitation occurred (Figure 4A). Moreover, the spectrogram displayed the EEG change in real-time, with changes in the depth of anesthesia (Figure 4B). Spectral bands of delta (1–4 Hz), theta (4–8 Hz), alpha (8–13 Hz), and beta (13–30 Hz) were analyzed from two groups (Figure 4C). Sevoflurane induced an increase in power within the 1–30 Hz (delta, theta, alpha and beta band) frequency ranges from awake (baseline) to deep anesthesia (LOTS and/or Nadir of Ai index). At LOTS and Nadir of Ai index, EEG signatures were characterized by generally high-amplitude and low-peak frequency, reflecting increased delta and decreased beta band activity. However, it exhibited a decrease in delta band and increases in alpha and beta bands during agitation. No differences in theta band were found. Additionally, we also calculated some other parameters of EEG for further study (Figure 4D). Anesthesia induction with sevoflurane was related to an increase in delta band (%), decreases in beta band (%), and spectral edge 95 (SEF95). During the occurrence of agitation, the fast waves (alpha and beta) were more pronounced, and the slow rhythms (delta) were less prominent, with higher median power frequency (MPF) (*p* < 0.001) and SEF95 (*p* < 0.01) compared to the nadir of Ai index.

**Figure 5:**
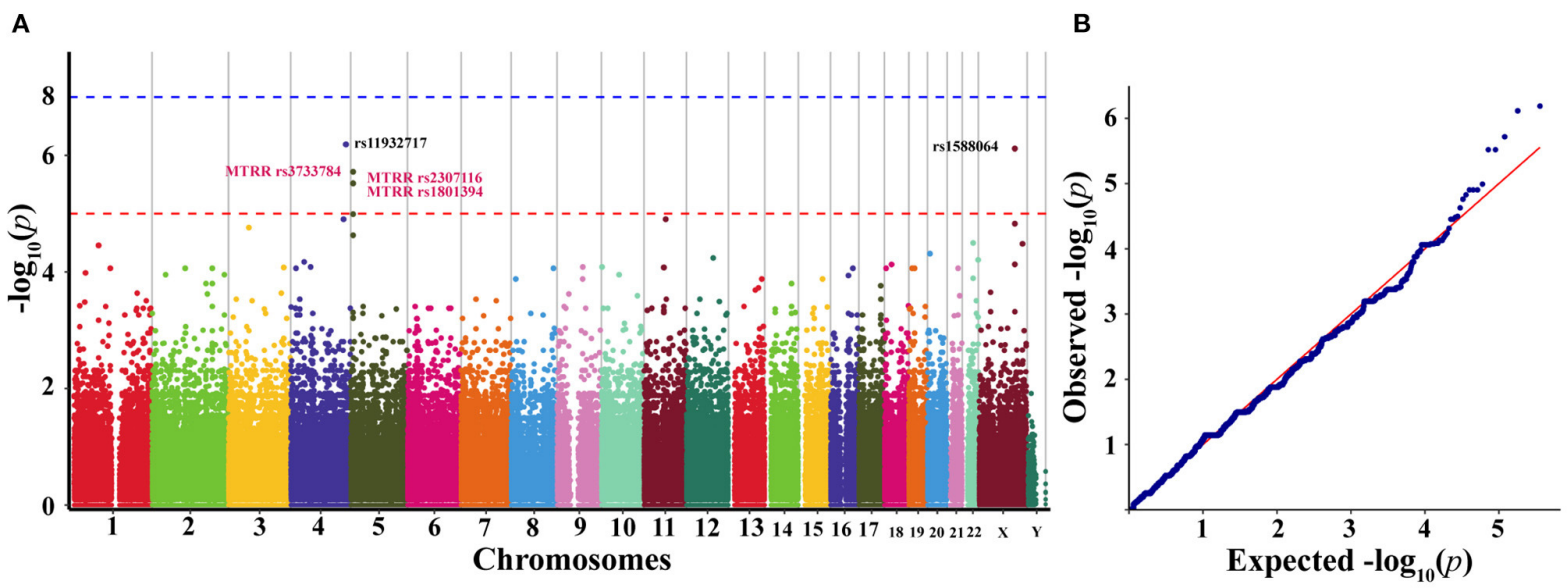
Manhattan plot **(A)** and quantile-quantile plot **(B)** representing the results of the genome-wide association study (GWAS) of the discovery cohort (*n* = 30). **(A)** Manhattan plot representing the association between SNPs and the susceptibility to agitation induced by sevoflurane anesthesia induction. The x-axis exhibits the chromosome number and y-axis exhibits genome-wide *p*-values. The genome-wide significance threshold of *p* = 5.0 × 10^−6^ is represented by the red horizontal line. **(B)** Quantile-quantile plot of genotyped and imputed SNPs. GWAS, genome-wide association study; SNPs, single-nucleotide polymorphisms.

Based on the results of GWAS (*n* = 30), we observed five SNPs displaying significant association with the susceptibility to agitation (defined by *p* < 5.0 × 10^−6^) (Table 2). Manhattan plot and quantile-quantile plot for the susceptibility to agitation were shown in Figure 5. Among these five SNPs, rs11932717, rs3733784, rs2307116, and rs1801394 came from genic regions (intronic or exonic regions). The remaining one, rs1588064, came from noncoding regions. Interestingly, rs3733784, rs2307116, and rs1801394 are in the MTRR gene. The most valuable association was observed for MTRR rs1801394 on chromosome five, which showed significant association (*p* = 3.02 × 10^−6^) with the susceptibility to agitation. Additionally, LD analysis revealed that these five SNPs were in moderate LD (*R*^2^ 0.33–0.70) (Table 2).

**Table 2:** SNPs displaying significant association with the susceptibility to agitation by GWAS (*n* = 30).

**CHR**	**SNP**	**Gene**	**Allele**	**Position**	***P*** **-value**	***R*** ^**2**^
4	rs11932717	/	A > C	175,950,601	6.50 × 10^−7^	0.68
X	rs1588064	/	G > A	115,892,959	7.68 × 10^−7^	0.33
5	rs3733784	MTRR	T > C	7,862,926	1.92 × 10^−6^	0.58
5	rs2307116	MTRR	G > A	7,861,985	3.02 × 10^−6^	0.70
5	rs1801394	MTRR	A > G	7,870,973	3.02 × 10^−6^	0.70

*GWAS, genome-wide association study; SNPs, single-nucleotide polymorphisms; CHR, chromosome; R^2^, Linkage Disequilibrium Analysis; MTRR, methionine synthase reductase*.

To verify the accuracy of the results of GWAS, we then sequenced these five SNPs using the SNaPshot technology in all patients (*n* = 176). Higher frequency of rs2307116 G > A (*p* = 0.009) and rs1801394 A > G (*p* = 0.006) were found in agitation (+) group compared to agitation (-) group (Table 3). Lower frequency of rs11932717 A > C (*p* < 0.001) and rs1588064 G > A (*p* = 0.025) were found in agitation (+) group compared to agitation (-) group. There was no difference in the frequency of rs3733784 T > C (*p* = 0.125) between the two groups.

**Table 3:** Distribution of SNPs associated with the susceptibility to agitation in all included patients (*n* = 176).

**SNP**	**Gene**	**Allele**	**Agitation (+)** ***n*** **(frequency)**	**Agitation (-)** ***n*** **(frequency)**	**Chi-square**	***P*** **-value**
rs11932717	/	AA	21 (0.84)	72 (0.48)	11.350	<0.001
		CC+AC	4 (0.16)	79 (0.52)		
rs1588064	/	GG	20 (0.80)	85 (0.56)	5.009	0.025
		AA+GA	5 (0.20)	66 (0.44)		
rs3733784	MTRR	TT	7 (0.28)	67 (0.44)	2.359	0.125
		CC+TC	18 (0.72)	84 (0.56)		
rs2307116	MTRR	GG	7 (0.28)	85 (0.56)	6.881	0.009
		AA+GA	18 (0.72)	66 (0.44)		
rs1801394	MTRR	AA	7 (0.28)	87 (0.58)	7.560	0.006
		GG+GA	18 (0.72)	64 (0.42)		

*Gene frequency were expressed as number (percentage). p < 0.05 was considered statistically significant*.

*SNPs, single-nucleotide polymorphisms; MTRR, methionine synthase reductase*.

To determine which variables could be used in agitation prediction, univariable logistic regression analysis was performed using the age, sex, BMI, rs11932717 A > C, rs1588064 G > A, rs3733784 T > C, rs2307116 G > A, and rs1801394 A > G as independent variables and experienced agitation as the dependent variable (Table 4). It indicated that the rs1801394 A > G (odds ratio 3.50, 95% CI 1.43–9.45, *p* = 0.008) and rs2307116 G > A (3.31, 1.36–8.95, *p* = 0.011) could be used as significant predictors of agitation. Carrying rs11932717 A > C (0.17, 0.05–0.48) and rs1588064 G > A (0.32, 0.10–0.84) may predict a lower risk of agitation. There was no obvious relationship between age, sex, BMI, and the risk of agitation.

**Table 4:** Univariable logistic regression analysis of preoperative variables and SNPs for predicting the susceptibility to agitation (*n* = 176).

**Variables**	**Univariable OR (95% CI)**	***P*** **-value**
**Baseline characteristics**		
Age, years	0.99 (0.95–1.03)	0.445
Male sex (*vs* female)	1.32 (0.56–3.12)	0.518
BMI, kg/m^2^	1.04 (0.90–1.19)	0.618
**SNPs**		
rs11932717 A > C	0.17 (0.05–0.48)	0.002
rs1588064 G > A	0.32 (0.10–0.84)	0.031
rs3733784 T > C	2.05 (0.84–5.54)	0.130
rs2307116 G > A	3.31 (1.36–8.95)	0.011
rs1801394 A > G	3.50 (1.43–9.45)	0.008

*Data are OR (95% CI). p < 0.05 was considered statistically significant*.

*OR, odds ratios; CI, confidence interval; BMI, body mass index; SNPs, single-nucleotide polymorphisms*.

## Discussion

In the present study, we discovered that (*i*) anesthesia induction with sevoflurane could induce agitation and its incidence was 14.2% (25/176); (*ii*) Patients with agitation were characterized as increases in Ai index, EMG, HR, and MAP; (*iii*) EEG displayed a shift toward high frequencies with spikes during agitation. The fast waves (alpha and beta) were more pronounced and the slow rhythms (delta) were less prominent; and (*iv*) we provided preliminary evidence of three SNPs in the MTRR gene of known folate metabolism function associated with the susceptibility to agitation.

In our study, sevoflurane induced a decrease in MAP in a dose-related manner, and HR remains nearly constant before the occurrence of agitation. These data are consistent with previous reports. All volatile anesthetics have an impact on the cardiovascular system, either through reducing systemic vascular resistance or by direct effects on the myocardium itself (5). Sevoflurane reduces the MAP and cardiac output index in a dose-dependent fashion (22). Previous studies of sevoflurane have not displayed an increase in HR in humans at clinically used concentrations as well (23, 24). However, agitation/paradoxical excitation during sevoflurane anesthesia induction exhibited a significant and rapid increase in HR and MAP in our study. This early increase in HR may result from the withdrawal of parasympathetic cardiac activity (5). We therefore speculate that both increases in HR and MAP may associate with inhibition of the parasympathetic and transitory relative increase in sympathetic vascular tone. Uncoordinated movements and increased muscle tension during agitation may also contribute to it. Additionally, increased muscle tone may cause an increase in EMG of frontal-temporal muscle. Clinically, rapid changes in hemodynamics (hypertension, tachycardia, etc.) may lead to an increment in the incidence of perioperative adverse events, especially in high-risk individuals with cardiovascular complications. Therefore, these issues should be paid close attention in the clinical work.

Different anesthetics induce distinct brain states through acting at different molecular targets and neural circuits, which are continuously visible in the EEG (25). Thus, brain states could be manifested by the characteristic changes in EEG activity in a relatively direct manner. Our findings are consistent with previous studies in which frontal power predominance increased with deeper sevoflurane anesthesia, including delta, theta, alpha, and beta band (26). Interestingly, in our study, the typical EEG patterns seen during agitation with sevoflurane anesthesia was marked by a distinct shift to high frequencies with spikes. These changes were accompanied by increases in the fast waves (alpha and beta) and decreases in the slow rhythms (delta). These results may demonstrate a relationship between the agitation and these changes in EEG. Agitation and changes of EEG both were simultaneous and transient in anesthesia induction. The rapid onset of cerebral effects of volatile anesthetics and different effects on the cortical and subcortical areas may contribute to it (27). The presence of spontaneous movement, including stiffening, blinking, limbs, and jaw movements accompanied with the high cerebral concentration of isoflurane (3–10%), and the EEG shown isoelectric with sharp transients (28). However, the isoelectric with sharp transients were not observed in patients with agitation in our study and the underlying mechanisms remain unknown. Moreover, anesthesia induction with sevoflurane may induce seizures or seizure-like activity (12, 29), but this was not the same as the EEG patterns observed in patients with agitation. There is no proof to recommend that the abnormal behaviors or high frequencies with spikes activities share same neural mechanisms as anesthetics-induced agitation/paradoxical excitation. Therefore, it may serve to emphasize the importance of further research to investigate the neurophysiological mechanisms regarding the generation of the EEG. In clinics, the monitoring of high frequencies with spikes patterns during anesthesia induction may provide the possibility to identify agitation in time.

Our clinical observations found that individuals showed difference in the susceptibility to agitation. Our results of GWAS found five SNPs exhibiting significant association with the susceptibility to agitation. LD analysis revealed that these five SNPs were in moderate LD (*R*^2^ 0.33–0.70). Interestingly, both rs3733784, rs2307116, and rs1801394 are SNPs in the MTRR gene involved in folate metabolism. Carrying rs1801394 A > G and/or rs2307116 G > A may predict a higher risk of agitation. MTRR plays an important role in the remethylation of homocysteinemia (Hcy) to methionine. In particular, MTRR rs1801394 has been related to abnormal plasma Hcy levels (30). It had been observed that individuals with the G allele of rs1801394 have an association with the higher risk of developing hyperhomocysteinemia (HHcy) (31). Neuronal excitotoxicity induced by Hcy has been repeatedly demonstrated (32–34). Hcy induced calcium influx through N-methyl-D-aspartate (NMDA) channel activation, which motivated glutamate neuronal excitotoxicity (34). Therefore, Hcy-induced neuronal excitotoxicity may underlie the susceptibility to agitation. Moreover, a recent study reported that general anesthesia can induce folate metabolism disorder and consequently demyelination in the developmental brain (35). Demyelination is related to motor deficits, and childhood exposure to multiple procedures requiring general anesthesia increases the risk of attention-deficit hyperactivity disorder (ADHD) (36–38). These data propose that general anesthesia may impede neuronal transportation and communication, causing motor deficits or abnormal movements like agitation/paradoxical excitation. The genetic screening of the MTRR gene before anesthesia may have the potential to assist the prediction of agitation/paradoxical excitation, avoid its occurrence. However, the complex relationship between agitation/paradoxical excitation and folate metabolism, and specific molecular mechanisms require further investigation. In addition, a previous study reported that GABR2 genetic polymorphisms may be involved in emergence agitation in pediatric anesthesia with sevoflurane (9). But this genetic polymorphism was not observed in our study using GWAS. One important reason may contribute to this discrepancy. Only GABARs genetic polymorphisms were selected and analyzed in the previous study. But in our study, a total of 359,584 high-quality SNPs were used to perform GWAS, and the SNaPshot technology was used for validation based on the results of GWAS. Therefore, contrasting with previous reports, our methods and results were more systematic, and by far more comprehensive.

There were several limitations in our study. Firstly, the EEG recordings analyzed in this study were obtained from frontal channels, and our analysis was unable to examine anterior-posterior connectivity and different brain regions dynamics. Thus, a wide-band multi-channel EEG recording is essential to analyze the different sources of EEG patterns. Secondly, there is an increasing recognition that GWAS results in too many false positives, especially with small sample sizes. We adopted a series of filtering strategies mentioned above and used the currently recognized linear model (GLM) in TASSEL v.5.2.54 for GWAS to avoid false positives as much as possible. Moreover, based on the results of GWAS, we sequenced these five SNPs in all included patients using the SNaPshot technology for validation. Thirdly, our study is associative rather than causative. The results of this study can only suggest correlations between SNPs and agitation, but cannot demonstrate causation. These SNPs that we found merit further definitive investigation through a larger sample size cohort study to increase statistical power and validate its relevance. Meanwhile, functional studies in mice models using genome editing methodologies should be carried out as well. Additionally, anesthesia induction with sevoflurane is most often used in pediatric anesthesia, and anesthesia-induced agitation in children is the most common and the most extensively investigated. But only adults in the age of 25–55 years were included in this study. Future studies should be adequately powered and consider age stratification.

In conclusion, our present study suggests that the agitation/paradoxical excitation induced by sevoflurane is characterized as increases in Ai index, EMG, HR, and MAP, and the high frequency with spikes in EEG. Moreover, our results provide preliminary evidence for MTRR genetic polymorphisms, involving folate metabolism function, may be related to the susceptibility to agitation.

## Data Availability Statement

The datasets presented in this study can be found in online repositories. The names of the repository/repositories and accession number(s) can be found at: https://doi.org/10.6084/m9.figshare.16832650.v1.

## Ethics Statement

The studies involving human participants were reviewed and approved by Institutional Ethics Committee of Tongji Medical College, Huazhong University of Science and Technology. The patients/participants provided their written informed consent to participate in this study.

## Author Contributions

XC had full access to all of the data in the study and take responsibility for the integrity of the data and the accuracy of the data analysis and obtained funding. SZ and XC: concept and design. SZ, LH, RZ, SH, YW, FX, SS, LX, and XC: acquisition, analysis, or interpretation of data. SZ and LH: drafting of the manuscript and statistical analysis. All authors contributed to the article and approved the submitted version.

## Funding

This work was supported by the National Key Research and Development Program of China (Grant 2018YFC2001802 to XC) and National Natural Science Foundation (Grant 82071251 to XC).

## Conflict of Interest

The authors declare that the research was conducted in the absence of any commercial or financial relationships that could be construed as a potential conflict of interest.

## Publisher's Note

All claims expressed in this article are solely those of the authors and do not necessarily represent those of their affiliated organizations, or those of the publisher, the editors and the reviewers. Any product that may be evaluated in this article, or claim that may be made by its manufacturer, is not guaranteed or endorsed by the publisher.
